# Mathematical modeling in municipal solid waste management: case study of Tehran

**DOI:** 10.1186/s40201-016-0250-2

**Published:** 2016-05-18

**Authors:** Mohsen Akbarpour Shirazi, Reza Samieifard, Mohammad Ali Abduli, Babak Omidvar

**Affiliations:** Department of Industrial Engineering and Management Systems, Amirkabir University of Technology, Tehran, Iran; Department of Environmental Engineering, Graduate Faculty of Environment, University of Tehran, Azin alley, Ghods St. Enghelab Ave., Tehran, Iran

**Keywords:** Mathematical modeling, Municipal solid waste management, Tehran, Lingo

## Abstract

**Background:**

Solid Waste Management (SWM) in metropolises with systematic methods and following environmental issues, is one of the most important subjects in the area of urban management. In this regard, it is regarded as a legal entity so that its activities are not overshadowed by other urban activities. In this paper, a linear mathematical programming model has been designed for integrated SWM. Using Lingo software and required data from Tehran, the proposed model has been applied for Tehran SWM system as a case study.

**Results:**

To determine the optimal status of the available system for Tehran’s Solid Waste Management System (SWMS), a novel linear programming model is applied. Tehran has 22 municipal regions with 11 transfer stations and 10 processing units. By running of the model, the transfer stations and processing units are decreased to 10 and 6 units, respectively.

**Conclusions:**

The proposed model is an alternative method for improvement the SWMS by decreasing the transfer stations and processing units.

## Background

Solid Waste Management (SWM) is a set of consistent and systematic regulations related to control generation, storage, collection, transportation, processing and landfilling of wastes according to the best public health principles, economy, preservation of resources, aesthetics, other environmental requirements and what the public attends to [1]. Many countries are facing problems in managing these problems and need comprehensive and practical solutions. Therefore, the optimization of conditions for sustainable approach to SWM is a key factor for managers and planners of government. Currently, the planners and decision-makers in the area of integrated SWM are confronting increased complexity, uncertainty, and multi-objective of this issue [2]. At the beginning, the process of decision-making on SWM was simple. It is because of the decisions were made only through simple comparison of some options out of the available options. However, different combinations of various components of this system were gradually propounded considering different factors in integrated SWM resulting in complexity of decision-making process.

At this period of time and regarding the complexities in the integrated solid waste management, decision-makers should distinguish between optimal, good, and fortuitous decision-making. In the optimal decision-making, one can solve the optimal problem using the techniques available in other fields. In this solution method, generally some constraints (criteria) are considered, where the function(s) is to be optimized through applying some methods. Good decision-making is done based on experience, trial and error or comparison between different options of the integrated SWM. Although it is possible to choose decisions close to the optimal state using this decision-making method, today these methods are not applicable due to increased number of different combinations in the decision-making process. In the fortuitous decision-making, since decisions are made with no scientific base, so the results are not acceptable [3].

In Iran, ever-increasing rate of population growth and constant development of cities, on the one hand, and proliferation and development of industrial, commercial, and service activities, on the other hand, have resulted in generation of large amounts of solid waste in cities. In the majority of cases, this has caused numerous problems considering shortage of facilities and budget. One of these problems is environmental pollution. Therefore, today a key factor of environmental pollution is mismanagement of different types of waste. Over 3.5 million tons of solid waste is generated daily around the world, where 80 % is recycled to the consumption cycle in developed countries and the rest is disposed or incinerated in a hygienic way [4].

The Tehran city is located in the north of Iran with a population of 8154051 (from the last census in 2011) [5], which currently generates around 6629 tons of municipal solid waste on a daily bases [6]. In 2004, with the support of the World Bank, extensive studies were initiated on the integrated SWM strategy of Tehran. The reports of these studies were finally delivered to the Organization of Waste Recycling & Composting (OWRC) in 2005 [7, 8]. However, the plan of integrated solid waste management in Tehran was never planned and pursued in the form of an executive program. The aim of presented study is the planning of the current status of SWM in Tehran with a scientific method.

The use of computational systems, due to absorption of a large number of resources and having great impacts on the environment, one can help decision-makers to achieve significant savings in costs and improve recovery of wastes [9]. Yu et al. [10] conducted a study on optimization of the long-term performance of municipal SWM system in the form of a bi-targeted mathematical model. In this paper, a linear and dynamic programming bi-targeted model is proposed for decision-making on supporting long-term operations of municipal SWM system. The proposed mathematical model simultaneously deals with calculation of the economic productivity and environmental pollution from the municipal SWM system within several time periods. Optimal exchange across the entire studied time horizon indicates the accuracy of this model. The proposed model has been calculated and solved by Lingo Software. The proposed model provided an effective solution for the long-term operational planning of the municipal SWMS.

Khaiwal et al. focused on the analysis of municipal SWM system and the methods to minimizing it in Chandigarh in India. This city is situated in the north part of India [11]. The information related to the SWM methods in Chandigarh was investigated for conductance of the mentioned research. The key information was collected from stakeholders through interviews and information of the registry related to transportation and solid waste disposal. This study has emphasized that the solid waste recycling system in this city is poor, where negligence of this important section has followed negative consequences. Thus, for environmentally-friendly SWM systems, a serious decision-making process and adjustment of operational activities are required. They suggested that the new frameworks should be framed on the properly design integrated solid waste management system with high recovery rates cost-effectiveness, and other environmental impacts.

Sie et al. [12] studied the optimal processing network for municipal SWM in Iskandar, Malaysia. In this paper, a mathematical programming model including integration of four principal consuming technologies has been presented to facilitate the optimal processing of the network. The mentioned model is able to predict the best combination out of the technologies of solid waste disposal, the procedure of solid waste recovery, prediction of product generation, estimation of the capacity of facilities, prediction of greenhouse gases (GHG) emitted from the system and eventually generation of the optimal and cost-effective solution for municipal SWM. The Mixed-Integer Linear Programming (MILP) model presented for profitability has been used as a model studied from the municipal SWM system. Based on results, the best mix of solid waste utilization technologies based on solid waste allocation to value added products was found to be landfill LFG capture (43.19 %), incineration (8.34 %), recycling (48.44 %) and composting (0.03 %).

Hui et al. [13] investigated the heat value of municipal solid waste in China considering the solid waste properties (physiochemical compounds). In this research, after investigation of the physiochemical properties of the municipal solid waste, the statistical indices including the mean, standard deviation, coefficient of changes, and physical analyses were used for determination of the heat value of municipal waste. The results showed that the chemical characteristics should be considered for thermal conversion process of Chinese SWM. Lohri et al. [14] examined the fiscal stability in municipal SWM, costs, and revenues of SWM in Bahir Dar City, Ethiopia. This research conducts a cost-income analysis based on data from July 2009 until June 2011. The analysis indicated that the overall costs in the SWM system in Bahir Dar has increased dramatically within this period due to increased costs related to the transportation of solid waste including the cost of solid waste collection from the households, commercial companies and institutions. The results of this research showed that existence of a structure for accurate analysis of costs and income from the SWM system was paramount to increase productivity and the cost and revenue balance in relation to cost. The obtained results revealed that a strong alliance between the municipality and private enterprise is an appropriate solution for improvement the financial sustainability of a SWM system.

Soltani et al. explored the numerous stakeholders in multi-criteria decision-making in municipal SWM. Municipal SWM is a complex process that includes several environmental, social, and economic criteria [15]. In addition, it also includes decision-making in solid waste management problems such as finding suitable sites for solid waste disposal or strategies commonly requiring various stakeholders such as governments, municipalities, industries, experts, and even the public. Results proved that the Analytical Hierarchy Process is the most common approach in consideration of multiple stakeholders.

Arena & Di Gregorio studies solid waste management planning based on analysis of the flow of the materials. This paper describes the results obtained from a municipal solid waste management planning [16]. This paper has investigated different components of SWM using analysis method of the flow of materials together with the results of the evaluation studies of the integrated solid waste management cycle. They found that the combination of material and substance flow analysis with an environmental assessment method is an alternative tool-box for comparing solid waste management technologies and scenarios.

Several models have been designed for integrated SWM systems. Although these models are a proper means for helping decision-makers and engineers in the integrated SWM planning process, each of them has considered only a certain portion of the SWM sections. In the present study, a mathematic model was used to optimize the current system of SWM in Tehran and identify the number of proper sites for transferring and processing of waste.

## Methods

In this research, a novel linear programming model is presented for investigation of the current status of municipal SWM in Tehran and for its optimization. This mathematical model tries to optimize the current system of SWM in Tehran and identify the number of proper sites for transferring and processing of waste. Furthermore, this model determines the extent of recovery and disposal of wastes using each of the recovery, landfilling, and composting methods. Another capability of the presented models is SWM based on the type and properties of the generated solid waste in every municipal region. Based on this ability, given the type of solid waste generated in the region, for each of them the best method of management is selected. The objective function in this model is increasing the profitability of the entire system of SWM.

**Fig. 1:**
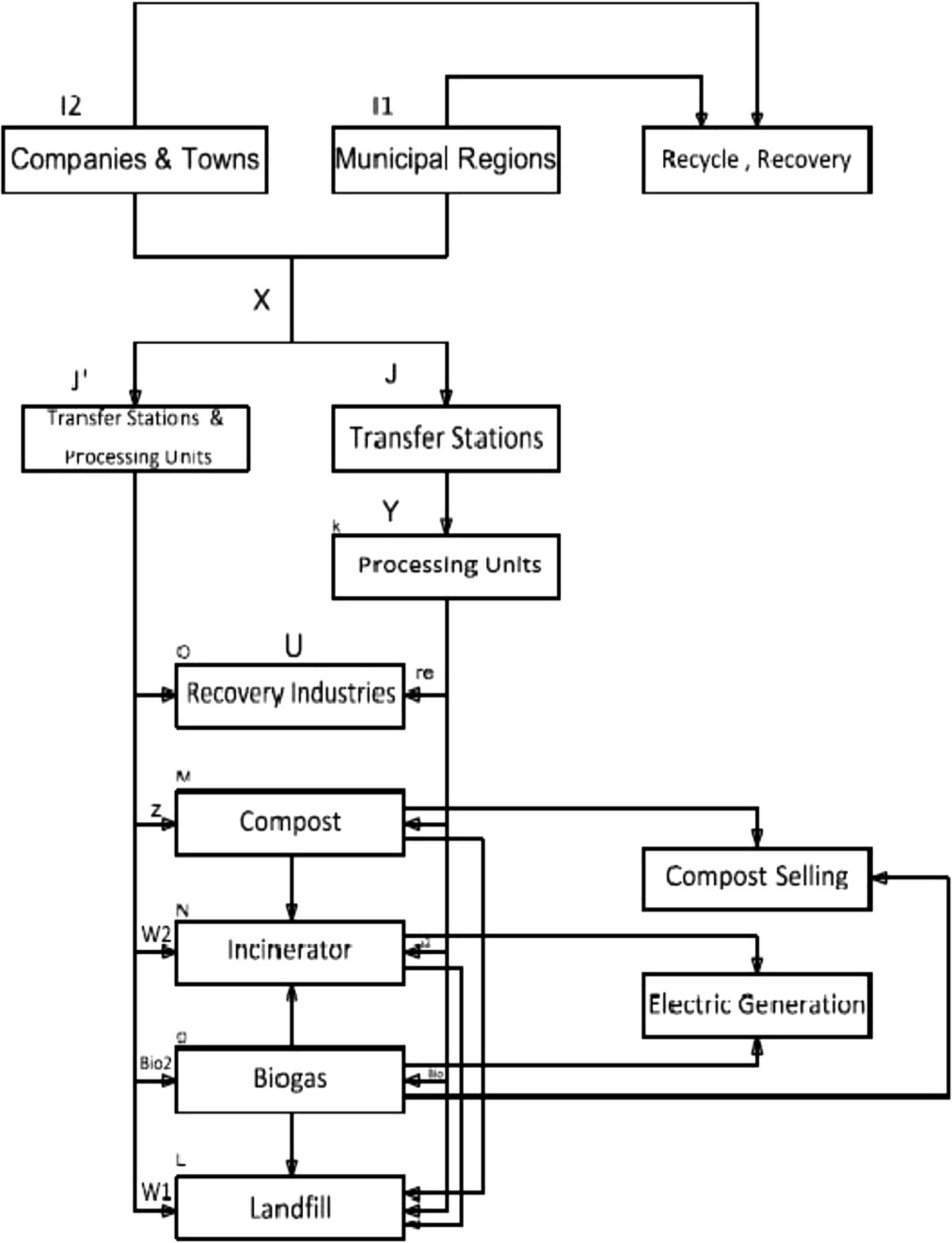
Conceptual representation of the model

Tehran municipality has placed presentation of urban services such as management and planning for organizing municipal wastes at the top of its agenda. Relevant officials of the solid waste recovery in 22 regions of Tehran were approached in order to collect data about the municipal solid waste generation through interviewing, filling out questionnaires, conducting field visits from Aradkooh landfilling and processing complex and collecting information on disposal and destiny of solid wastes. Based on the available data, Tehran’s wastes can be categorized into three groups of municipal wastes, organizational wastes together with the wastes generated by towns, hospitals, and animals. The municipality of each region is responsible for collection and transportation of the wastes generated in that region. The major part of the municipal wastes along with a part of the organizational and town wastes, are transferred to the transfer stations available throughout Tehran after collection. The wastes transferred to transfer stations are transported to the Aradkooh landfilling and processing complex, situated 32 km away from the south east of Tehran. The following conceptual representation is used as a base for Tehran municipal SWM modeling (Fig. 1).

### Conceptual representation of the model

As can be observed, the regions are connected only to the transfer stations. Under the current state, no processing is carried out at the transfer stations, but it can be provided in the future in these stations, accordingly, this property has been considered in the presented model. At the next stage, after collection of wastes at the transfer stations, the wastes are transported to other units called processing units to be processed. In these units, after processing and separation of wastes into three groups of dry valuable, dry non-valuable and wet wastes, they are transferred to other units. The separated solid waste is guided to one of the recovery, composting, or landfilling stages based on the type of developed optimal productivity. The proposed model has the ability of separating and allocating wastes to the mentioned units, bringing about the maximum profitability. Furthermore, based on the policies of SWM development in Tehran, a number of other facilities including incinerator, biogas, and electricity generation have also been considered in the model. Locating of the transfer stations and processing units is another property of this model. Accordingly, in this paper, a linear mathematical programming model is developed based on the solid waste flow process in Tehran. The list of symbols, parameters and variables concerning the mathematical model has been shown in Appendix I.

### Model constraints

In case Y_j_ = 1 therefore the station j will be established as a transfer station and if the Y_j_^'^ = 1 therefore the station j will be established as the transfer station and processing unit:

1 $$ {\mathrm{Y}}_{\mathrm{j}}+{\mathrm{Y}}_{\mathrm{j}}^{\mathit{\hbox{'}}}\ \le 1 $$

Each region connects to one station:

2 $$ {\displaystyle {\sum}_j{x}_{ij}+{\displaystyle {\sum}_j{x}_{ij}^{\mathit{\hbox{'}}}=1}} $$

If Y_j_ = 1, the *x*_*ij*_ can get value:

3 $$ {x}_{ij}\le {Y}_j $$

If Y_j_^'^ = 1, the *x*_*ij*_^'^ can get value:

4 $$ {x}_{ij}^{\mathit{\hbox{'}}}\le {Y}_j^{\mathit{\hbox{'}}} $$

The capacity constraint of transfer station j: the amount of unseparated (mixed) solid waste from region i to transfer station j has to less than the capacity of that station:

5 $$ {\displaystyle {\sum}_{\mathrm{i}}{D}_i\left(1-{\alpha}_i\right){x}_{ij}\le Ca{p}_{\mathrm{j}}^{\mathrm{s}}\kern0.5em \forall \mathrm{j}=1,2,\dots } $$

The capacity constraint of transfer station and processing unit j: the amount of unseparated (mixed) solid waste from region i to transfer station and processing unit j has to less than the capacity of that station:

6 $$ {\displaystyle {\sum}_{\mathrm{i}}{D}_i\left(1-{\alpha}_i\right){x}_{ij}^{\mathit{\hbox{'}}}\le Ca{p}_{\mathrm{j}}^{\mathit{\hbox{'}}\mathrm{s}}\kern0.5em \forall \mathrm{j}=1,2,\dots } $$

If the j is transfer station, the transferred solid waste from the regions will be sent to the processing units:

7 $$ {D}_i\left(1-{\alpha}_i\right){x}_{ij}={\displaystyle {\sum}_K{Y}_{ijK}} $$

The capacity of the processing units has to more than the incoming solid waste from transfer stations:

8 $$ {\displaystyle {\sum}_i{\displaystyle {\sum}_j{Y}_{ijK}\le Ca{p}_K^{pr}L{K}_k}} $$

The allocated solid waste to the transfer station and processing unit j, after transformation to waste P_2_, will be sent to one of the compost, recovery, biogas, incinerator or landfill facilities:

9 $$ {\displaystyle {\sum}_{P_1}{\displaystyle {\sum}_i{D}_i\left(1-{\alpha}_i\right){x}_{i{j}_1}^{\mathit{\hbox{'}}}{\delta}_{i{P}_1}{\gamma}_{P_1{P}_2}={\displaystyle {\sum}_M{\beta}_{P_2}^1{Z}_{\mathrm{jM}{P}_2}^2+{\displaystyle {\sum}_g{\beta}_{P_2}^5{\mathrm{Bio}}_{\mathrm{jg}{P}_2}^1+{\displaystyle {\sum}_{\mathrm{O}}{\beta}_{P_2}^4r{e}_{jO{P}_2}+{\displaystyle {\sum}_N{\beta}_{P_2}^3{W}_{3jN{P}_2}+{\displaystyle {\sum}_L{\beta}_{P_2}^2{W}_{4jL{P}_2}}}}}}}} $$

In the following, the left side of the equation shows the amount of dry valuable waste, dry non-valuable solid waste and wet solid waste after the processing unit. The flow of the mentioned wastes after the processing units could be the incinerator, landfill, compost, recovery, biogas facilities or combination of these facilities:

10 $$ {\displaystyle {\sum}_{P_1}{\displaystyle {\sum}_j{\displaystyle {\sum}_i{Y}_{ijK}{\delta}_{i{P}_1}{\gamma}_{P_1{P}_2}={\displaystyle {\sum}_N{\beta}_{P_2}^3{W}_{2KN{P}_2}+{\displaystyle {\sum}_L{\beta}_{P_2}^2{W}_{1KL{P}_2}+{\displaystyle {\sum}_M{\beta}_{P_2}^1{Z}_{KM{P}_2}+{\displaystyle {\sum}_{\mathrm{O}}{\beta}_{P_2}^4{U}_{KO{P}_2}+{\displaystyle {\sum}_g{\beta}_{P_2}^5{\mathrm{Bio}}_{\mathrm{Kg}{P}_2}^2}}}}}}}} $$

### The constraints relating to the capacity of facilities

Capacity of recovery facilities:

11 $$ {\displaystyle {\sum}_j{\displaystyle {\sum}_{P_2}r{e}_{jO{P}_2}+}}{\displaystyle {\sum}_K{\displaystyle {\sum}_{P_2}{U}_{KO{P}_2}\le Ca{p}_O^1\times L{O}_O\ \forall O}} $$

Capacity of compost facilities:

12 $$ {\displaystyle {\sum}_{P_2}{\displaystyle {\sum}_j{Z}_{\mathrm{jM}{P}_2}^2+{\displaystyle {\sum}_K{\displaystyle {\sum}_{P_2}{Z}_{KM{P}_2}\le Ca{p}_{\mathrm{M}}^2\times L{M}_M\ \forall M}}}} $$

Capacity of landfill facilities:

13 $$ {\displaystyle {\sum}_j{\displaystyle {\sum}_{P_2}{W}_{4jL{P}_2}+{\displaystyle {\sum}_N{\mathrm{SL}}_{\mathrm{NL}}^2+{\displaystyle {\sum}_L{\mathrm{SL}}_{\mathrm{ML}}^1+{\displaystyle {\sum}_K{\displaystyle {\sum}_{P_2}{W}_{1KL{P}_2}+{\displaystyle {\sum}_g{\mathrm{SL}}_{\mathrm{gL}}^3\le Ca{p}_{\mathrm{L}}^4\times L{L}_L\ \forall L}}}}}}} $$

Capacity of incinerator facilities:

14 $$ {\displaystyle {\sum}_j{\displaystyle {\sum}_{P_2}{W}_{3jN{P}_2}+{\displaystyle {\sum}_K{\displaystyle {\sum}_{P_2}{W}_{2KN{P}_2}+{\displaystyle {\sum}_M{\mathrm{IN}}_{\mathrm{MN}}^1+{\displaystyle {\sum}_g{\mathrm{IN}}_{gN}^2\le Ca{p}_{\mathrm{N}}^3\times L{N}_N\ \forall N}}}}}} $$

Capacity of biogas facilities:

15 $$ {\displaystyle {\sum}_j{\displaystyle {\sum}_{P_2}{\mathrm{Bio}}_{\mathrm{jg}{P}_2}^1+{\displaystyle {\sum}_{P_2}{\displaystyle {\sum}_K{\mathrm{Bio}}_{\mathrm{Kg}{P}_2}^2\le Ca{p}_{\mathrm{g}}\times L{g}_g\ \forall g}}}} $$

### Mass balance constraints

Mass balance relating to the compost facilities:

16 $$ {\displaystyle {\sum}_j{\displaystyle {\sum}_{P_2}{Z}_{\mathrm{jM}{P}_2}^2+{\displaystyle {\sum}_K{\displaystyle {\sum}_{P_2}{Z}_{KM{P}_2}=F{E}_{\mathrm{M}}^1+}}}}{\displaystyle {\sum}_{\mathrm{L}}{\mathrm{SL}}_{\mathrm{M}\mathrm{L}}^1+{\displaystyle {\sum}_{\mathrm{N}}{\mathrm{IN}}_{\mathrm{M}\mathrm{N}}^1}} $$

Mass balance relating to the biogas facilities:

17 $$ {\displaystyle {\sum}_j{\displaystyle {\sum}_{P_2}{\mathrm{Bio}}_{\mathrm{jg}{P}_2}^1+{\displaystyle {\sum}_{P_2}{\displaystyle {\sum}_K{\mathrm{Bio}}_{\mathrm{Kg}{P}_2}^2={\mathrm{EL}}_{\mathrm{g}}^1+F{E}_g^2+{\displaystyle {\sum}_N{\mathrm{IN}}_{gN}^2+{\displaystyle {\sum}_L{\mathrm{SL}}_{\mathrm{g}\mathrm{L}}^3}}}}}} $$

Mass balance relating to the incinerator facilities:

18 $$ {\displaystyle {\sum}_j{\displaystyle {\sum}_{P_2}{W}_{3jN{P}_2}+}}{\displaystyle {\sum}_{P_2}{\displaystyle {\sum}_K{W}_{2KN{P}_2}+}}{\displaystyle {\sum}_g{\mathrm{IN}}_{gN}^2+{\displaystyle {\sum}_M{\mathrm{IN}}_{\mathrm{MN}}^1={\mathrm{EL}}_{\mathrm{N}}^2+{\displaystyle {\sum}_{\mathrm{L}}{\mathrm{SL}}_{\mathrm{N}\mathrm{L}}^2}}} $$

### Environmental constraints

Environmental constraints relating to the transfer station:

19 $$ FE{A}_{jpe}{\displaystyle {\sum}_i{D}_i\left(1-{\alpha}_i\right)\left({x}_{ij}^{\mathit{\hbox{'}}}+{x}_{ij}\right)\le Vca{p}_{pe}} $$

Environmental constraints relating to the processing unit(s):

20 $$ FE{m}_{kpe}{\displaystyle {\sum}_i{\displaystyle {\sum}_j{Y}_{ijK}\le Mca{p}_{pe}}} $$

Environmental constraints relating to the compost facilities:

21 $$ FE{w}_{mpe}\left({\displaystyle {\sum}_k{\displaystyle {\sum}_{p_2}{Z}_{km{p}_2}+{\displaystyle {\sum}_{p_2}{\displaystyle {\sum}_j{Z}_{mj{p}_2}^2}}}}\right)\le Wca{p}_{pe} $$

Environmental constraints relating to the landfill facilities:

22 $$ VL\left({\displaystyle {\sum}_k{\displaystyle {\sum}_{p_2}{W}_{1 kL{p}_2}\times V{L}_{p2}+{\displaystyle {\sum}_j{\displaystyle {\sum}_{p_2}{W}_{4jL{p}_2}\times V{L}_{p2}}}}}\right)\le Scap $$

### Objective function

23 $$ max\ \left( benefit- cost\right) $$

24 $$ \begin{array}{c}\mathrm{cost}={\displaystyle {\sum}_{\mathrm{i}}{\displaystyle {\sum}_{\mathrm{j}}{\mathrm{D}}_{\mathrm{i}}\left(1-{\upalpha}_{\mathrm{i}}\right)\left({\mathrm{x}}_{\mathrm{i}\mathrm{j}} + {\mathrm{x}}_{\mathrm{i}\mathrm{j}}^{\mathit{\hbox{'}}}\right){\mathrm{C}}_{\mathrm{i}\mathrm{j}}^1+}{\displaystyle {\sum}_{\mathrm{j}}{\displaystyle {\sum}_{\mathrm{K}}{\displaystyle {\sum}_{\mathrm{i}}{\mathrm{Y}}_{\mathrm{i}\mathrm{j}\mathrm{K}}{\mathrm{C}}_{\mathrm{j}\mathrm{K}}^2+}}}{\displaystyle {\sum}_{{\mathrm{P}}_2}{\displaystyle {\sum}_{\mathrm{j}}{\displaystyle {\sum}_{\mathrm{O}}{\mathrm{re}}_{{\mathrm{j}\mathrm{O}\mathrm{P}}_2}{\mathrm{C}}_{\mathrm{j}\mathrm{O}}^3+}}}}{\displaystyle {\sum}_{{\mathrm{P}}_2}{\displaystyle {\sum}_{\mathrm{K}}{\displaystyle {\sum}_{\mathrm{O}}{\mathrm{U}}_{{\mathrm{K}\mathrm{O}\mathrm{P}}_2}{\mathrm{C}}_{\mathrm{K}\mathrm{O}}^4}}}\\ {}+{\displaystyle {\sum}_{{\mathrm{P}}_2}{\displaystyle {\sum}_{\mathrm{K}}{\displaystyle {\sum}_{\mathrm{M}}{\mathrm{Z}}_{{\mathrm{K}\mathrm{M}\mathrm{P}}_2}{\mathrm{C}}_{\mathrm{K}\mathrm{M}}^5+}}}{\displaystyle {\sum}_{{\mathrm{P}}_2}{\displaystyle {\sum}_{\mathrm{j}}{\displaystyle {\sum}_{\mathrm{M}}{\mathrm{Z}}_{{\mathrm{j}\mathrm{M}\mathrm{P}}_2}^2{\mathrm{C}}_{\mathrm{j}\mathrm{M}}^6+}}}{\displaystyle {\sum}_{\mathrm{j}}{\displaystyle {\sum}_{\mathrm{N}}{\displaystyle {\sum}_{{\mathrm{P}}_2}{\mathrm{W}}_{3{\mathrm{j}\mathrm{N}\mathrm{P}}_2}{\mathrm{C}}_{\mathrm{j}\mathrm{N}}^7+}}}{\displaystyle {\sum}_{\mathrm{K}}{\displaystyle {\sum}_{{\mathrm{P}}_2}{\displaystyle {\sum}_{\mathrm{N}}{\mathrm{W}}_{2{\mathrm{K}\mathrm{N}\mathrm{P}}_2}{\mathrm{C}}_{\mathrm{K}\mathrm{N}}^7}}}\\ {}+{\displaystyle {\sum}_{\mathrm{M}}{\displaystyle {\sum}_{\mathrm{N}}{\mathrm{IN}}_{\mathrm{M}\mathrm{N}}^1{\mathrm{C}}_{\mathrm{M}\mathrm{N}}^8+}}{\displaystyle {\sum}_{\mathrm{N}}{\displaystyle {\sum}_{\mathrm{g}}{\mathrm{IN}}_{\mathrm{g}\mathrm{N}}^2{\mathrm{C}}_{\mathrm{g}\mathrm{N}}^9+}}{\displaystyle {\sum}_{{\mathrm{P}}_2}{\displaystyle {\sum}_{\mathrm{j}}{\displaystyle {\sum}_{\mathrm{g}}{\mathrm{Bio}}_{{\mathrm{j}\mathrm{g}\mathrm{P}}_2}^1{\mathrm{C}}_{\mathrm{g}\mathrm{j}}^{10}+}}}{\displaystyle {\sum}_{{\mathrm{P}}_2}{\displaystyle {\sum}_{\mathrm{K}}{\displaystyle {\sum}_{\mathrm{g}}{\mathrm{Bio}}_{{\mathrm{K}\mathrm{g}\mathrm{P}}_2}^2{\mathrm{C}}_{\mathrm{K}\mathrm{g}}^{11}}}}\\ {}+{\displaystyle {\sum}_{\mathrm{M}}{\displaystyle {\sum}_{\mathrm{L}}{\mathrm{SL}}_{\mathrm{M}\mathrm{L}}^1{\mathrm{C}}_{\mathrm{M}\mathrm{L}}^{12}+}}{\displaystyle {\sum}_{\mathrm{N}}{\displaystyle {\sum}_{\mathrm{L}}{\mathrm{SL}}_{\mathrm{N}\mathrm{L}}^2{\mathrm{C}}_{\mathrm{N}\mathrm{L}}^{13}+}}{\displaystyle {\sum}_{\mathrm{g}}{\displaystyle {\sum}_{\mathrm{L}}{\mathrm{SL}}_{\mathrm{g}\mathrm{L}}^3{\mathrm{C}}_{\mathrm{g}\mathrm{L}}^{14}+}}{\displaystyle {\sum}_{{\mathrm{P}}_2}{\displaystyle {\sum}_{\mathrm{K}}{\displaystyle {\sum}_{\mathrm{L}}{\mathrm{W}}_{1{\mathrm{K}\mathrm{L}\mathrm{P}}_2}{\mathrm{C}}_{\mathrm{K}\mathrm{L}}^{15}+}}}{\displaystyle {\sum}_{{\mathrm{P}}_2}{\displaystyle {\sum}_{\mathrm{j}}{\displaystyle {\sum}_{\mathrm{L}}{\mathrm{W}}_{4{\mathrm{j}\mathrm{L}\mathrm{P}}_2}{\mathrm{C}}_{\mathrm{j}\mathrm{L}}^{16}}}}\\ {}+{\displaystyle {\sum}_{\mathrm{o}}{\mathrm{C}\mathrm{O}}_{\mathrm{o}}{\mathrm{L}\mathrm{O}}_{\mathrm{o}}+}{\displaystyle {\sum}_{\mathrm{k}}{\mathrm{C}\mathrm{K}}_{\mathrm{k}}{\mathrm{L}\mathrm{K}}_{\mathrm{k}}+}{\displaystyle {\sum}_{\mathrm{m}}{\mathrm{C}\mathrm{M}}_{\mathrm{m}}{\mathrm{L}\mathrm{M}}_{\mathrm{m}}+}{\displaystyle {\sum}_{\mathrm{l}}{\mathrm{C}\mathrm{L}}_{\mathrm{l}}{\mathrm{L}\mathrm{L}}_{\mathrm{l}}+}{\displaystyle {\sum}_{\mathrm{n}}{\mathrm{C}\mathrm{N}}_{\mathrm{n}}{\mathrm{L}\mathrm{N}}_{\mathrm{n}}+}{\displaystyle {\sum}_{\mathrm{g}}{\mathrm{C}\mathrm{g}}_{\mathrm{g}}{\mathrm{L}\mathrm{g}}_{\mathrm{g}}+{\mathrm{C}\mathrm{j}}_{\mathrm{j}}^1{\mathrm{Y}}_{\mathrm{j}}+{\mathrm{C}\mathrm{j}}_{\mathrm{j}}^2{\mathrm{Y}}_{\mathrm{j}}^{\mathit{\hbox{'}}}}\\ {}+\left({\displaystyle {\sum}_{\mathrm{M}}{\displaystyle {\sum}_{\mathrm{L}}{\mathrm{SL}}_{\mathrm{M}\mathrm{L}}^1+}}{\displaystyle {\sum}_{\mathrm{N}}{\displaystyle {\sum}_{\mathrm{L}}{\mathrm{SL}}_{\mathrm{N}\mathrm{L}}^2+}}{\displaystyle {\sum}_{\mathrm{g}}{\displaystyle {\sum}_{\mathrm{L}}{\mathrm{SL}}_{\mathrm{g}\mathrm{L}}^3+}}{\displaystyle {\sum}_{\mathrm{K}}{\displaystyle {\sum}_{\mathrm{L}}{\displaystyle {\sum}_{\mathrm{P}2}{\mathrm{W}}_{\mathrm{K}\mathrm{L}\mathrm{P}2}^1+}}}{\displaystyle {\sum}_{\mathrm{j}}{\displaystyle {\sum}_{\mathrm{L}}{\displaystyle {\sum}_{\mathrm{P}2}{\mathrm{W}}_{\mathrm{j}\mathrm{L}\mathrm{P}2}^4}}}\right)\mathrm{Cdis}\end{array} $$

25 $$ Benefit=\left({\displaystyle {\sum}_{P_2}\left({\displaystyle {\sum}_j{\displaystyle {\sum}_Or{e}_{jO{P}_2}+}}{\displaystyle {\sum}_K{\displaystyle {\sum}_O{U}_{KO{P}_2}}}\right)}{B}_{P_2}^1\right) + {\alpha}_i{D}_i{B}^2+{\displaystyle {\sum}_g{\mathrm{EL}}_{\mathrm{g}}^1{B}_g^3+{\displaystyle {\sum}_N{\mathrm{EL}}_{\mathrm{N}}^2{B}_N^4+}}{\displaystyle {\sum}_MF{E}_{\mathrm{M}}^1{B}_M^5+{\displaystyle {\sum}_gF{E}_g^2{B}_g^6}} $$

### Solving model with Lingo software

The process of solving a math program requires a large number of calculations and is, therefore, best performed by a computer program. Lingo is a mathematical modeling language designed particularly for formulating and solving a wide variety of optimization problems including linear programing. Lingo optimization software uses branch and bound methods to solve problems of this type. The obtained model is solved by Lingo 13.0 optimization software.

## Results and discussion

### Case study

The presented model has been applied for a case study related to the SWM system in Tehran. Tehran contains 22 regions in which the amount of daily municipal solid waste generation is 6629 tons. Moreover, the wastes generated in the regions by citizens and organizations have been categorized in 20 different groups. These groups are further converted to three groups of dry valuable (type 1), dry non-valuable (type 2) and wet (type 3) wastes at processing units. Table 1 provides the amount of generated solid waste in the regions as well as its composition in terms of percentage in 2014 [6]. The second column of the mentioned table indicates the amount of daily generated solid waste (in terms of ton). The third column itself and onward indicate the ratio of each of the solid waste types out of the total generated solid waste (in terms of percentage).

**Table 1:** Amount and composition of municipal solid waste generated in regions in 2014

Region no	Waste amount	Used bread	Plastic	PET	Plaster	Talc	Foam	Paper	Cardboard	Ferrous metal	Non-ferrous metal	Textile	Glass	Wood	Rubber	Soil	Special	Putrescible material	Other
1	448	0.04	0.04	0.02	0.02	0.01	0.00	0.08	0.08	0.01	0.01	0.00	0.03	0.00	0.00	0.00	0.01	0.65	0.00
2	516	0.04	0.03	0.01	0.05	0.01	0.00	0.08	0.03	0.02	0.01	0.01	0.03	0.00	0.00	0.00	0.03	0.65	0.00
3	305	0.02	0.03	0.02	0.04	0.00	0.00	0.07	0.04	0.01	0.00	0.01	0.01	0.01	0.00	0.00	0.02	0.69	0.00
4	703	0.05	0.03	0.01	0.04	0.01	0.00	0.04	0.06	0.02	0.00	0.01	0.02	0.01	0.01	0.01	0.02	0.69	0.00
5	534	0.04	0.03	0.01	0.03	0.01	0.00	0.07	0.04	0.01	0.01	0.01	0.02	0.01	0.00	0.00	0.03	0.68	0.00
6	278	0.03	0.03	0.01	0.03	0.00	0.00	0.11	0.05	0.01	0.01	0.01	0.03	0.00	0.00	0.00	0.05	0.62	0.01
7	278	0.05	0.03	0.01	0.03	0.00	0.00	0.06	0.03	0.03	0.01	0.01	0.04	0.00	0.00	0.01	0.04	0.66	0.00
8	273	0.05	0.03	0.02	0.03	0.01	0.00	0.06	0.04	0.02	0.00	0.03	0.02	0.00	0.00	0.00	0.02	0.68	0.00
9	130	0.05	0.04	0.01	0.04	0.01	0.00	0.05	0.04	0.03	0.00	0.02	0.02	0.02	0.00	0.01	0.03	0.62	0.00
10	202	0.04	0.02	0.01	0.02	0.01	0.01	0.04	0.04	0.03	0.01	0.03	0.01	0.03	0.03	0.02	0.06	0.59	0.01
11	247	0.06	0.03	0.02	0.02	0.00	0.01	0.05	0.03	0.04	0.00	0.01	0.02	0.00	0.00	0.00	0.01	0.71	0.00
12	339	0.03	0.04	0.02	0.03	0.00	0.00	0.06	0.05	0.02	0.00	0.02	0.01	0.00	0.00	0.00	0.04	0.68	0.01
13	163	0.05	0.02	0.01	0.04	0.01	0.00	0.05	0.04	0.03	0.01	0.01	0.02	0.00	0.00	0.01	0.04	0.65	0.00
14	321	0.04	0.02	0.01	0.02	0.00	0.00	0.04	0.07	0.02	0.00	0.01	0.01	0.00	0.00	0.00	0.01	0.75	0.00
15	470	0.06	0.02	0.01	0.04	0.01	0.00	0.05	0.04	0.03	0.00	0.02	0.02	0.00	0.00	0.00	0.03	0.68	0.00
16	229	0.06	0.01	0.01	0.04	0.01	0.00	0.03	0.05	0.02	0.00	0.01	0.01	0.00	0.00	0.01	0.04	0.71	0.00
17	183	0.05	0.03	0.01	0.03	0.01	0.00	0.05	0.03	0.02	0.00	0.01	0.01	0.00	0.00	0.00	0.01	0.73	0.00
18	282	0.03	0.03	0.01	0.02	0.01	0.00	0.06	0.01	0.02	0.00	0.01	0.02	0.00	0.01	0.02	0.02	0.73	0.00
19	200	0.05	0.03	0.02	0.04	0.00	0.00	0.04	0.04	0.02	0.00	0.02	0.01	0.00	0.00	0.00	0.04	0.69	0.00
20	309	0.03	0.01	0.01	0.03	0.00	0.00	0.03	0.03	0.02	0.00	0.01	0.02	0.00	0.01	0.01	0.03	0.77	0.00
21	119	0.05	0.03	0.02	0.04	0.01	0.00	0.06	0.04	0.03	0.01	0.01	0.01	0.00	0.01	0.00	0.02	0.67	0.00
22	100	0.04	0.02	0.01	0.03	0.01	0.00	0.05	0.04	0.03	0.00	0.02	0.02	0.00	0.00	0.00	0.01	0.71	0.00

### Amount and composition of municipal solid waste generated in regions in 2014

**Fig. 2:**
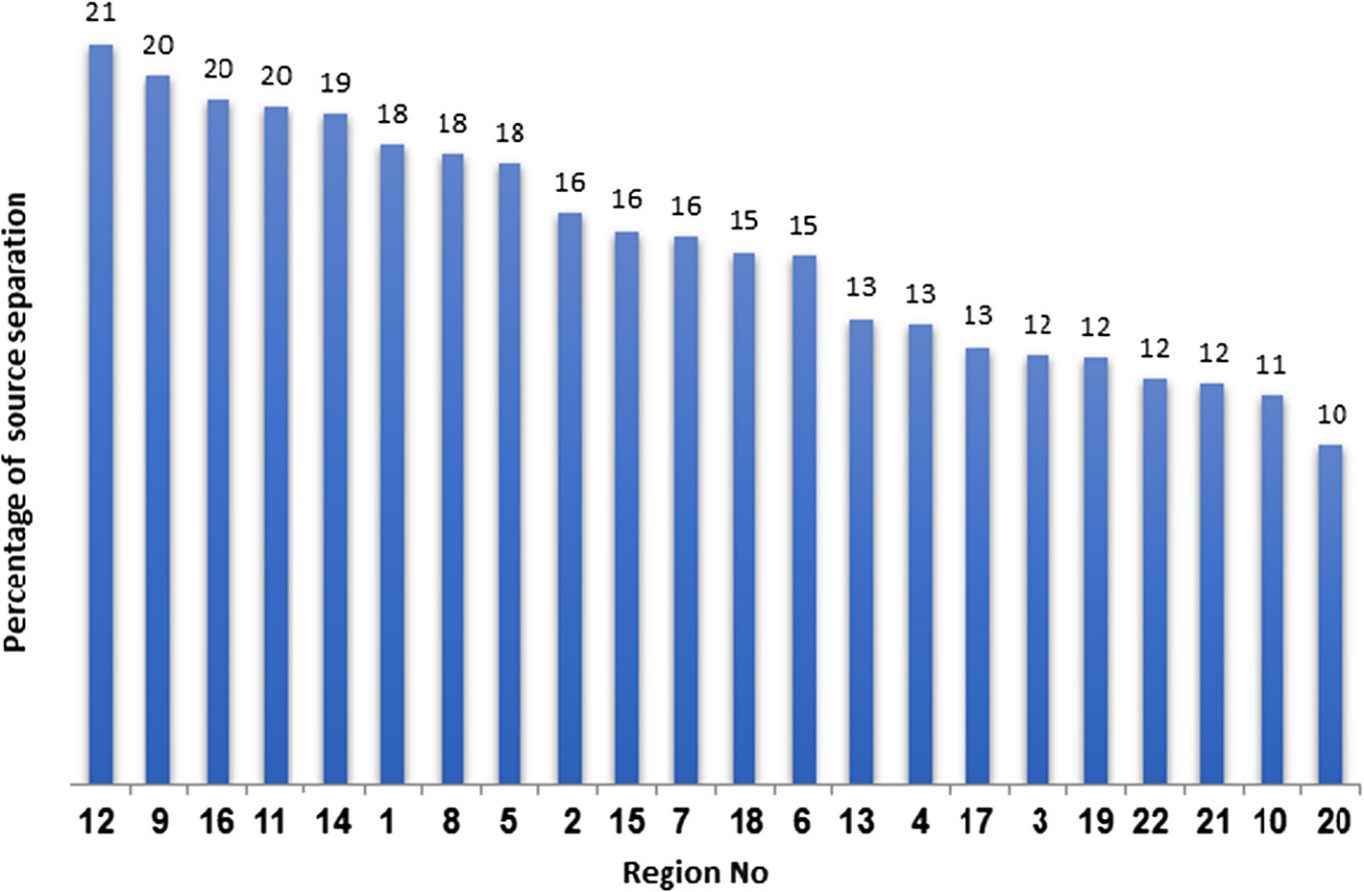
Status of solid waste separation at source for each region in 2014

Furthermore, some of the solid waste generated in the point of origin and before the collection are separated. These wastes are called dry solid waste separated at source. The following figure represents the percentage of solid waste separated at source for every region [6] (Fig. 2).

### Status of solid waste separation at source for each region in 2014

There are 11 transfer stations in the SWM system of Tehran. In all of these 11 stations, only collection and transfer of solid waste take place with no processing operation. Table 2 shows the nominal capacity of all of these stations and the current status of the regions allocated to them together with the amount of solid waste sent to them [6].

**Table 2:** Specifications of municipal solid waste transfer stations in Tehran in 2014

Transfer station name	Covered region(s)	Nominal capacity (ton/day)	Receiving waste (ton/day)
Darabad	1,3	1299	540–570
Zanjan	2,10	1037	1000
Banihashem	4,8	485	400–450
Hakimiyeh	4,8	485	780–790
Kuhak	5,21,22	2227	750
Beyhaghi	3,6,7	864	750–800
Harandi	11,12	691	600–650
Azadegan	13,14,15	1484	1100
Yaran	9,10,17,18	1484	700
Jehad	16,19	1484	500–530
Shahid Avini	20	323	250–300

### Specifications of municipal solid waste transfer stations in Tehran in 2014

**Fig. 3:**
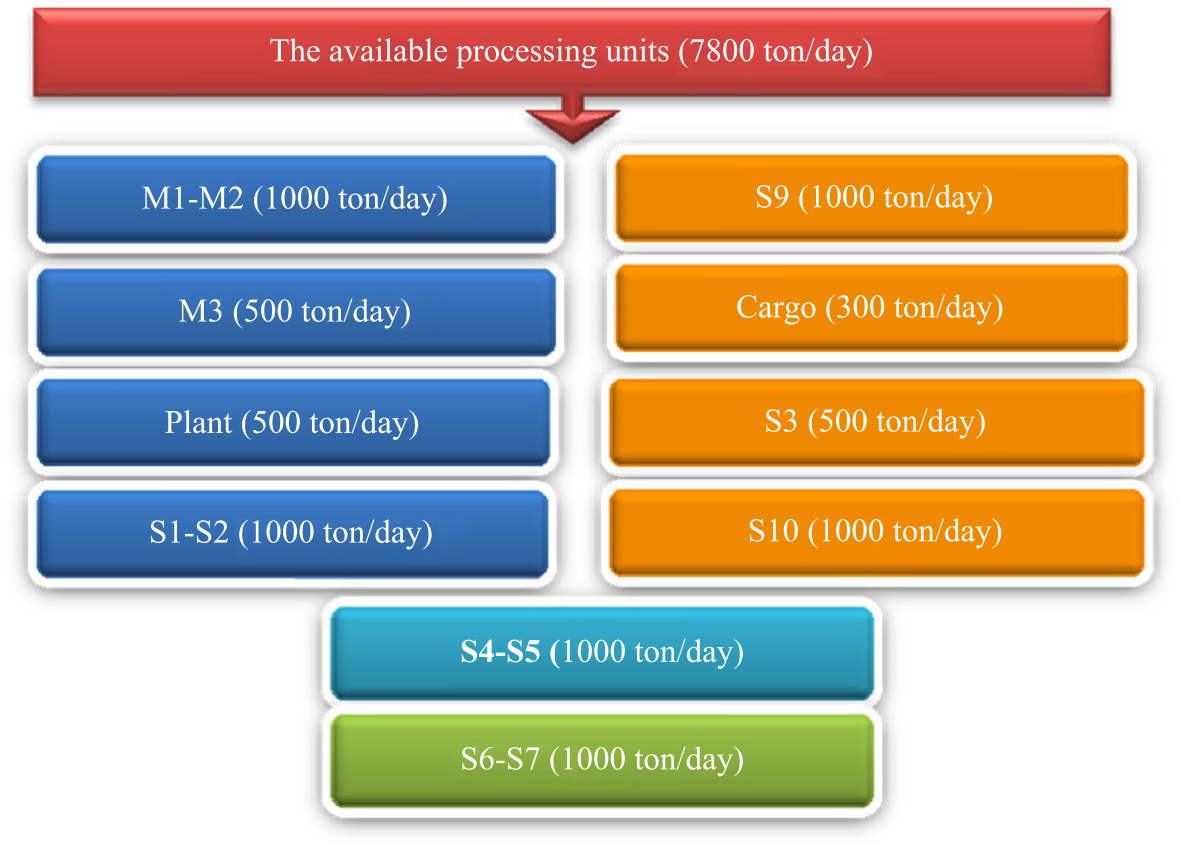
Name and nominal capacity of solid waste processing units in Aradkooh

On the other hand, in Tehran’s SWM system, there are 10 solid waste processing units available, all of which are located in the Aradkooh landfilling and processing complex in Tehran. All of the collected wastes are transferred to this complex. The following figure indicates the solid waste processing units’ capacity (in terms of ton) per day [6] (Fig. 3).

### Name and nominal capacity of solid waste processing units in Aradkooh

In the current situation, there is no possibility for generation of electricity using biogas and incineration facilities, since these facilities are still under construction. Therefore, only generation of compost, recovery of dry materials and landfilling of solid waste are done in the Aradkooh complex. Currently, there are two compost generation facilities with the capacities of 2200 and 1800 tons/day, one center of solid waste recovery with a capacity of 50000 tons/day, and one solid waste landfilling center with a capacity of 60000 tons/day are available.

In order to determine the optimal status of the available system for Tehran’s SWM system, after extraction of information and run of the model, the following results were obtained. The results indicated a cost amounting to 577179 US $. It means in the best scenario, the existing system incurs a cost of 577179 US $ per day for the municipal SWM. Accordingly, the current facilities and systems are unprofitable.

The results obtained from the optimization of the current situation indicate that all of the 11 available transfer stations are not processing units and they act in the form of transfer stations only. Therefore, no region is directly connected to the processing units and the solid waste of all of the 22 regions of Tehran is first moved to transfer stations, and then goes to the processing units. The following table summarizes the way the 22 regions of Tehran are covered by transfer stations as output of the model. As can be seen, the transfer station 3 (Banihashem) is established neither as a transfer nor a processing center. Thus the transfer station 3 can be closed based on Lingo optimization results (Table 3).

**Table 3:** Model result relating the regions’ allocation to the transfer stations (X_ij_)

Transfer station name	Region(s) to be covered
Darabad	1
Zanjan	2
Banihashem	-
Hakimiyeh	8
Kuhak	5,21,22
Beyhaghi	3,6,7
Harandi	11,12,16
Azadegan	13,14,15
Yaran	9,10,17,18
Jehad	4,19
Shahid Avini	20

### Model result relating the regions’ allocation to the transfer stations (X_ij_)

As output of the model, the amount of solid waste allocated from each region to the processing unit is shown in Table 4. The first column in this table represents the name of the available processing units, while the other columns indicate the name of the region allocated to the processing unit and the amount of solid waste sent to them. Based on the obtained results, the processing units of M3, cargo, Plant, and S3 should not be reopened. Moreover, five different regions send their wastes to the processing units of S10 and M1-M2.

**Table 4:** Model result concerning regions’ solid waste allocation to the processing units

Processing unit	Regions to be covered									
	Region	Waste amount	Region	Waste amount	Region	Waste amount	Region	Waste amount	Region	Waste amount
M1-M2	3	226.3	4	107	12	267.8	17	159.2	18	239.7
S9	2	433.4	4	164.7	13	141.8	14	260	-	-
M3	-	-	-	-	-	-	-	-	-	-
Cargo	-	-	-	-	-	-	-	-	-	-
Plant	-	-	-	-	-	-	-	-	-	-
S3	-	-	-	-	-	-	-	-	-	-
S1-S2	1	367.3	11	158.6	22	88	20	278.1	-	-
S10	6	236.3	7	233.5	8	223.9	9	104	10	179.8
S4-S5	3	42	15	394.8	19	176	21	104.7	-	-
S6-S7	4	339.9	5	437.9	11	39	16	183.2	-	-

### Model result concerning regions’ solid waste allocation to the processing units

Table 5 indicates that each transfer station is allowed to send its collected solid waste to which processing unit. Some of the transfer stations are allowed to transfer their wastes to multiple processing units such as the transfer station 6 connected to three processing units.

**Table 5:** Model result concerning transfer stations’ allocation to the processing units

Processing unit	Allocated transfer stations
M1-M2	6,7,9,10
S9	2,8,10
M3	-
Cargo	-
Plant	-
S3	-
S1-S2	1,5,7,11
S10	4,6,9
S4-S5	6,8,5,10
S6-S7	5,7,10

### Model result concerning transfer stations’ allocation to the processing units

Furthermore, two composts, landfilling, and recovery facilities are also established. The amount of compost generated from the compost facility 1 is 2200 tons and from facility 2 is 1800 tons. As previously stated, the solid waste Type 1 is not allowed to be sent to the compost facility and thus is directly transferred to the recovery facilities, where only solid waste of Type 2 and 3 are sent to the compost facilities. Meanwhile, the processing units of 1 and 2 and 7–10 are also opened. Furthermore, most of the solid waste Type 2 has been transferred to the landfilling facility and landfilled, where solid waste Type 3 has been sent for compost. The whole solid waste of the Type 1 has been sent to the recovery facilities and recovered. Table 6 indicates the amount of different types of wastes sent from different processing units to the compost, landfill and recovery facilities.

**Table 6:** Model result concerning solid waste flow after the processing units

Facility name	Waste type	M1-M2	S9	S1-S2	S10	S4-S5	S6-S7
Compost No.1	Dry non-valuable (Type 2)	-	-	-	-	-	187.3
	Wet (Type 3)	702	682	628.2	-	-	-
Compost No.2	Type 2	-	-	-	-	-	-
	Type 3	-	-	-	621.8	488.3	689.9
Landfill	Type 2	33.4	28.6	15.2	42.6	23.8	31.6
	Type 3	-	-	-	-	-	-
Recovery	Dry valuable (Type 1)	264.7	289.5	248.6	313	205.5	278.5

### Model result concerning solid waste flow after the processing units

At the end, the results obtained from the model solution indicate the decreased number of the available transfer stations and processing units. From among the transferring stations, station 3 has the potential to be closed due to proximity to the other transfer stations. Similarly, the processing units of M1-M2, S9, S1-S2, S10, S4-S5 and S6-S7 have the potential to respond to the solid waste sent by the transfer stations, thus there is no need to reopen other processing units. The amount of solid waste sent to the compost facilities of 1 and 2 is equal to their entire capacity necessitating them to operate with their full capacity. Therefore, the combination of optimal transfer and processing units can generate the most acceptable daily solid waste collection and transport cycle. Recent studies indicated that adopting integrating solid waste treatment technologies like composting with effective source separation for organic fraction, solid waste recovery or recycling can help in achieving economic and environmental benefits [17]. Sharholy et al. reviewed the municipal solid waste management in Indian cities. They found that the management of MSW requires proper infrastructure, maintenance and upgrade for four activities, i.e., solid waste generation, collection, transportation, and disposal [18]. However, the optimization of SWM due to consideration of the interaction of various factors (e.g. economic development, social policy, environmental quality) and parameters (e.g. operation cost, transportation cost, and waste- generation rate), is difficult [19]. Therefore, multi- criteria decision analysis (MCDA) is an alternative method to optimize the MSWM. Xi et al. used constrained mixed-integer linear programming (ICCMILP) method. There are 6 transfer stations with the total capacity of 5500 ton/d, charged with the classification and pretreatment of MSW so as to increase the utilization rate and volume reduction efficiency. It was estimated that through compression and dehydration, most of the leachate would be removed, and the compression ratio would be 2.4–4 times of solid waste compactors.

## Conclusion

In this paper, a linear programming model has been presented for optimization of the transferring and processing units of solid waste in Tehran. Tehran has 22 municipal regions in which 20 types of solid waste are generated. Similarly, there are 11 transfer stations and 10 processing units in Tehran. After running of the model, the results have indicated that the optimal situation for Tehran in order to maximize the profitability was presence of 10 transfer station and 6 processing units. Furthermore, the amount of solid waste sent to the compost facilities of 1 and 2 is equal to their entire capacity necessitating them to operate with their full capacity. Future improvement can be focused on two aspects. First, determination of physic- chemical composition of solid waste in Tehran to predict the proximate and ultimate analysis and heating value from physical composition. Second, the optimization of incineration, recycling and composting after completing facilities in Tehran.

## Appendix 1 List of symbols and variables

### Indices

**i:** region’s solid waste (I1) and companies and industrial parks (I2)

**j:** urban service station (transfer station)

**K:** processing unit

**L:** Landfill unit

**M:** compost

**N:** incinerator

**O:** Recovery industries

**P**_**1**_**:** Solid waste type

**P**_**2**_**:** dry valuable waste, dry non- valuable waste, wet waste

**g:** biogas

**pe:** different types of environmental pollutions

### Parameters and Variables

*Cap*_j_^s^: urban service station capacity (transfer station)

*Cap*_j_^' s^: urban service station capacity, in case the station is the transfer station and processing unit

*α*_*i*_: percentage of the solid waste of the region i recovered directly (source separation)

*D*_*i*_: the amount of solid waste generated in the region i

${\delta}_{i{P}_1}$: the amount (percentage) of solid waste P_1_ generated in the region i

*Cap*_*K*_^*pr*^_:_ the capacity of the processing unit K

${\gamma}_{P_1{P}_2}$: the amount (percentage) of solid waste P_1_ transformed into solid waste P_2_${\displaystyle {\sum}_{P_2}{\gamma}_{P_1{P}_2}=1}$

${\beta}_{P_2}^1$: if the solid waste P_2_ sent to the compost the value will be 1, otherwise 0

${\beta}_{P_2}^2$: if the solid waste P_2_ sent to the landfill the value will be 1, otherwise 0

${\beta}_{P_2}^3$: if the solid waste P_2_ sent to the incinerator the value will be 1, otherwise 0

${\beta}_{P_2}^4$: if the solid waste P_2_ sent to the recovery the value will be 1, otherwise 0

${\beta}_{P_2}^5$: if the solid waste P_2_ sent to the biogas the value will be 1, otherwise 0

*Cap*_g_: the capacity of the biogas g

*Cap*_*O*_^1^: the capacity of the recovery O

*Cap*_M_^2^: the capacity of the compost M

*Cap*_N_^3^: the capacity of the incinerator N

*Cap*_L_^4^: the capacity of the landfill L

*FEA*_*jpe*_: the amount of pe generated in transfer station j

*Vcap*_*pe*_: the maximum allowed pe in transfer station j

*Mcap*_*pe*_: the maximum allowed pe in processing unit

*Wcap*_*pe*_: the maximum allowed pe in compost unit

Scap: the maximum allowed Leachate in landfill unit

*FEm*_*kpe*_: the amount of pe generated in processing unit K

*FEw*_*mpe*_: the amount of pe generated in compost unit M

*VL*_*p*2_: the amount of generated Leachate as per landfilled solid waste P_2_

*x*_*ij*_: if the solid waste of region i allocated to transfer station j the value is 1, otherwise 0

*x*_*ij*_^'^: if the solid waste of region i allocated to transfer and processing station j the value is 1, otherwise 0

*Y*_*ijK*_: the amount of the demand of the solid waste of region i accumulated in the transfer station j and sent to the processing unit K

${W}_{1KL{P}_2}$: the amount of solid waste P_2_ sent from processing unit K to landfill L

${W}_{2KN{P}_2}$: the amount of solid waste P_2_ sent from processing unit K to incinerator N

${W}_{3jN{P}_2}$: the amount of solid waste P_2_ sent from transfer and processing station j to incinerator N

${W}_{4jL{P}_2}$: the amount of solid waste P_2_ sent from transfer and processing station j to landfill L

${Z}_{KM{P}_2}$: the amount of solid waste P_2_ sent from processing unit K to compost M

${U}_{KO{P}_2}$: the amount of solid waste P_2_ sent from processing unit K to recovery O

${Z}_{\mathrm{jM}{P}_2}^2$: the amount of solid waste P_2_ sent from transfer station j to compost M

${\mathrm{Bio}}_{\mathrm{jg}{P}_2}^1$: the amount of solid waste P_2_ sent from transfer station j to biogas g

$r{e}_{jO{P}_2}$: the amount of solid waste P_2_ sent from transfer station j to recovery O

*φ*_*i*_: fraction of generated solid waste in region i to be separated in source

${\mathrm{Bio}}_{\mathrm{Kg}{P}_2}^2$: the amount of solid waste P_2_ sent from processing unit K to biogas g

*FE*_M_^1^: the amount of the generated compost in compost M

*FE*_*g*_^2^: the amount of the generated compost in biogas g

EL_g_^1^: the amount of the generated electricity in biogas g

EL_N_^2^: the amount of the generated electricity in incinerator N

SL_ML_^1^: the amount of the solid waste (compost) sent from compost M to landfill L

SL_NL_^2^: the amount of the solid waste (ash) sent from incinerator N to landfill L

SL_gL_^3^: the amount of the solid waste sent from biogas g to landfill L

IN_MN_^1^: the amount of the solid waste (compost) sent from compost M to incinerator N

IN_*gN*_^2^: the amount of the solid waste sent from biogas g to incinerator N

*Lg*_*g*_: 1 = if the biogas g selected, otherwise = 0

*LK*_*k*_: 1 = if the processing K selected, otherwise = 0

*LN*_*N*_: 1 = if the incinerator N selected, otherwise = 0

*LL*_*L*_: 1 = if the landfill L selected, otherwise = 0

*LM*_*M*_: 1 = if the compost M selected, otherwise = 0

*LO*_*O*_: 1 = if the recovery O selected, otherwise = 0

*Y*_*j*_: in case j is transfer station

*Y*_j_^'^: in case j is transfer station and processing unit

*C*_*ij*_^1^: transfer cost from region i to transfer station j

*C*_*jK*_^2^: transfer cost from transfer station j to processing unit K

*C*_*jO*_^3^: transfer cost from transfer station j to recovery industry O

*C*_*KO*_^4^: transfer cost from processing unit K to recovery industry O

*C*_*KM*_^5^: transfer cost from processing unit K to compost M

*C*_*Mj*_^6^: transfer cost from transfer station j to compost M

*C*_*jN*_^7^: transfer cost from transfer station j to incinerator N

*C*_*MN*_^8^: transfer cost from compost M to incinerator N

*C*_*gN*_^9^: transfer cost from biogas g to incinerator N

*C*_*gj*_^10^: transfer cost from transfer station j to biogas g

*C*_*Kg*_^11^: transfer cost from processing unit K to biogas g

*C*_*ML*_^12^: transfer cost from compost M to landfill L

*C*_*NL*_^13^: transfer cost from incinerator N to landfill L

*C*_*gL*_^14^: transfer cost from biogas g to landfill L

*C*_*KL*_^15^: transfer cost from processing unit K to landfill L

*C*_*jL*_^16^: transfer cost from transfer station j to landfill L

*Cdis*: landfill cost per unit waste

*CO*_*o*_: establishment cost of recovery O

*CK*_*k*_: establishment cost of processing unit K

*CM*_*m*_: establishment cost of compost M

*CL*_*l*_: establishment cost of landfill L

*CN*_*n*_: establishment cost of incinerator N

*Cg*_*g*_: establishment cost of biogas g

$C{j}_{j{\mathrm{Y}}_{\mathrm{j}}}^1$: establishment cost of transfer station j

$C{j}_{j{\mathrm{Y}}_{\mathrm{j}}^{\hbox{'}}}^2$: establishment cost of transfer station and processing unit j

${B}_{P_2}^1$: the benefit from the recovery of one unit of solid waste type P_2_

*B*^2^: the benefit from the separated solid waste at source

*B*_*g*_^3^: the benefit from the sell of one unit of generated electricity in biogas g

*B*_*N*_^4^: the benefit from the sell of one unit of generated electricity in incinerator N

*B*_*M*_^5^: the benefit from the sell of one unit of compost from compost M

*B*_*g*_^6^: the benefit from the sell of one unit of compost from biogas g
